# The effects of almond consumption on fasting blood lipid levels: a systematic review and meta-analysis of randomised controlled trials

**DOI:** 10.1017/jns.2016.19

**Published:** 2016-08-16

**Authors:** Kathy Musa-Veloso, Lina Paulionis, Theresa Poon, Han Youl Lee

**Affiliations:** Intertek Scientific and Regulatory Consultancy, 2233 Argentia Road, Suite 201, Mississauga, Ontario, L5N 2X7, Canada

**Keywords:** Almonds, Blood lipids, Cholesterol, TAG, CAD, coronary artery disease, HDL-C, HDL-cholesterol, LDL-C, LDL-cholesterol, T2DM, type 2 diabetes mellitus, TC, total cholesterol

## Abstract

A systematic review and meta-analysis of randomised controlled trials was undertaken to determine the effects of almond consumption on blood lipid levels, namely total cholesterol (TC), LDL-cholesterol (LDL-C), HDL-cholesterol (HDL-C), TAG and the ratios of TC:HDL-C and LDL-C:HDL-C. Following a comprehensive search of the scientific literature, a total of eighteen relevant publications and twenty-seven almond-control datasets were identified. Across the studies, the mean differences in the effect for each blood lipid parameter (i.e. the control-adjusted values) were pooled in a meta-analysis using a random-effects model. It was determined that TC, LDL-C and TAG were significantly reduced by −0·153 mmol/l (*P* < 0·001), −0·124 mmol/l (*P* = 0·001) and −0·067 mmol/l (*P* = 0·042), respectively, and that HDL-C was not affected (−0·017 mmol/l; *P* = 0·207). These results are aligned with data from prospective observational studies and a recent large-scale intervention study in which it was demonstrated that the consumption of nuts reduces the risk of heart disease. The consumption of nuts as part of a healthy diet should be encouraged to help in the maintenance of healthy blood lipid levels and to reduce the risk of heart disease.

Almonds are nutritionally dense^(^^1^^)^. According to compositional data from the United States Department of Agriculture, 100 g of raw, unroasted almonds provides 2423 kJ (579 kcal), 50 g of fat, 13 g of insoluble dietary fibre and 21 g of protein^(^^2^^)^. There is some natural variability in the composition of almonds in terms of the fat and fatty acid contents; when expressed on a per 100 g basis, almonds contain about 45 to 54 g of fat, with relative amounts of PUFA, MUFA and SFA of 9 to 15, 25 to 36, and 3 to 5 g, respectively^(^^1^^)^. In addition, almonds contain small amounts of plant sterols^(^^3^^–^^5^^)^, and are sources or high sources of several minerals and vitamins (i.e. Ca, Fe, Mg, P, K, Zn, Cu, Mg, thiamin, riboflavin, niacin and vitamin E), according to the requirements for nutrition claims, as set out in Regulation EC 1924/2006^(^^6^^)^.

Several of the compositional attributes of almonds are ideal for the maintenance of healthy blood lipid levels. Indeed, in a meta-analysis of randomised controlled trials, Phung *et al.*^(^^7^^)^ reported that almond consumption was associated with a significant reduction in total cholesterol (TC) (−0·18 mmol/l; 95 % CI −0·34, −0·02 mmol/l), as well as a strong trend toward a reduction in LDL-cholesterol (LDL-C) (−0·15 mmol/l; 95 % CI −0·29, 0·00 mmol/l). No effects on HDL-cholesterol (HDL-C), TAG, or on the ratio of LDL-C:HDL-C were observed. The meta-analysis by Phung *et al.*^(^^7^^)^ was based on five randomised controlled studies (representing a total of 142 participants). More than 7 years have elapsed since Phung *et al.*^(^^7^^)^ conducted their literature search; thus, all randomised controlled trials that have since been published were identified, and, using the totality of evidence, the effects of almonds on blood lipid levels were re-evaluated.

## Methods

### Literature search

The systematic review was conducted in accordance with the guidelines of the Preferred Reporting Items for Systematic Reviews and Meta-analyses (PRISMA) statement.

In May 2013, the electronic search tool Dialog™ was used to search eight literature databases (Allied & Complementary Medicine™, Adis Clinical Trials Insight, CAB Abstracts, Elsevier Biobase, EMBASE^®^, Foodline^®^: Science, Medline^®^, and National Technical Information Service). Dialog™ was subsequently replaced by ProQuest Dialog™, which did not index one of the eight original literature databases that were searched (i.e. Elsevier Biobase). Updated literature searches were conducted in May 2014 and in February 2015, using ProQuest Dialog™ to access seven of the eight original databases.

The search terms used reflected the exposure (‘almond’, ‘*Prunus amygdalus*’, ‘*P. amygdalus*’, ‘*Prunus dulcis*’, ‘*P. dulcis*’) and the study population (‘human’, ‘subject’, ‘participant’, ‘volunteer’, ‘patient’, ‘elder*’, ‘senior’, ‘geriatric’, ‘adult’, ‘men’, ‘women’, ‘man’, ‘woman’, ‘teen*’, ‘adolescen*’, ‘people’, ‘person’, ‘individual’), and were required to appear in the titles or abstracts of the articles. The literature searches were restricted to studies conducted in humans; that is, the keywords used to limit the searches to human studies (‘animal’, ‘rodent’, ‘rat’, ‘mouse’, ‘mice’, ‘dog’, ‘pig’, ‘rabbit’, ‘hamster’, ‘monkey’, ‘*in vitro*’, ‘*ex vivo*’) were required not to appear in the descriptor or subject field of the records. No restrictions with respect to the health outcomes of interest or language were imposed on any of the literature searches. As well, no restriction on the year of publication was imposed on the first literature search.

### Inclusion/exclusion criteria

The following inclusion criteria were applied: (1) a human intervention study that was randomised and controlled (such that the control group/phase could have consisted of either no food or any food(s) without tree nuts or fractions of tree nuts); (2) a full-length article that was published in a peer-reviewed journal; (3) the objective of the study (either primary or secondary) was to assess the effects of almond consumption on blood lipid levels; (4) the amount of almonds consumed was reported; (5) the subjects were adults (aged ≥18 years of age) without serious disease such as heart disease; (6) the study duration was ≥4 weeks; (7) fasting blood lipids (i.e. TC, LDL-C, HDL-C and/or TAG) were assessed; and (8) fasting blood lipids were measured using validated methods.

The following exclusion criteria were applied: (1) the publication was of a secondary research study (e.g. systematic review or meta-analysis); (2) the objective of the study (either primary or secondary) was not to assess the effects of almond consumption on blood lipid levels; (3) the subjects had a serious disease (e.g. heart disease, cancer) and were not representative of the general population; (4) the subjects were children or pregnant or lactating women; (5) control-adjusted effects on blood lipids could not be calculated from the data provided; (6) the independent effects of almonds on blood lipid levels could not be isolated (e.g. almonds were co-consumed with other nuts or another nutritional or pharmaceutical intervention); and (7) the study results for the same population group were published in another journal (i.e. the study was a kin publication to another study).

### Data extraction and study quality

Study data were extracted independently by two reviewers (T. P. and H. Y. L.), and the consistency of the two datasets was verified by a third reviewer (K. M. V.). Where there were inconsistencies or discrepancies between the two datasets, the original publication was consulted, and a consensus was reached via discussions between the three reviewers (T. P., H. Y. L. and K. M. V.). Data extracted from the studies included study design, country of study conduct, sample size, study population (proportion of males, health status, mean age, mean BMI, mean baseline blood lipid levels (i.e. TC, LDL-C, HDL-C, TAG, TC:HDL-C; LDL-C:HDL-C)), dietary interventions (dose, form of almonds, provision of foods or meals, duration, frequency of intake, pattern of intake), background diets and macronutrient intakes, statistical results, and the mean difference in the effect for each blood lipid parameter (see the Statistical analysis section for details on how the mean difference in the effect was calculated for the crossover and parallel studies).

Health Canada's standardised quality appraisal tool^(^^8^^)^ was used to determine the quality of the studies. A quantitative score (zero or one) was assigned to each of the fifteen items included in the tool, and studies with scores of ≥8/15 were considered to be ‘higher quality’ while studies with scores of ≤7/15 were considered to be ‘lower quality’. Study quality was appraised by one reviewer (L. P.).

### Statistical analysis

Several of the identified studies had multiple comparisons (e.g. one study may have had three arms, including one control and two different almond doses). Each almond-control comparison, hereinafter referred to as a stratum, was considered a separate trial; however, the control sample size was divided evenly amongst the comparisons so as to avoid inflating the weight of each stratum. For parallel studies, the mean difference in the effect for each blood lipid parameter was calculated as the change from baseline in the control group subtracted from the change from baseline in the almond group. For crossover studies, the mean difference in the effect for each blood lipid parameter was calculated as the blood lipid value at the end of the control phase subtracted from the blood lipid value at the end of the almond phase.

In order to determine the effects of almonds on each of the blood lipid parameters, the results of the studies were pooled in a meta-analysis, with the mean difference in the effect and the inverse of the variance used as the weighting factor. In the majority of the studies, variances for the mean differences were not reported; thus, variances were calculated using information provided in the publication (e.g. using CI or individual variances for the almond and control groups). If, in parallel studies, variances for the changes from baseline were reported separately for the almond and control groups, then a pooled variance for the mean difference was calculated. If, for parallel studies, variances only for the baseline and end of treatment values were reported, then these were used to calculate the variance for the change from baseline, using a correlation coefficient of 0·8. Similarly, for crossover studies, if variances only for the end of treatment values were reported, then the variance for the mean difference was calculated using a correlation coefficient of 0·8. A correlation coefficient of 0·8 was used because this value approximated that calculated from the studies in which variances were provided for the baseline, end of treatment, and change from baseline measures^(^^9^^–^^11^^)^. A random-effects model was used, according to the methods described by DerSimonian & Laird^(^^12^^)^, given that random-effects models take into consideration the variability in response both within and between studies.

The pooled estimates and accompanying 95 % CI were determined using Comprehensive Meta-analysis Software (version 2.2.064). Publication bias was assessed according to the trim-and-fill method developed by Duval & Tweedie^(^^13^^)^. With this method, asymmetry in the funnel plot is searched for. If the asymmetry is determined to be due to the presence of small studies (with large variances) in which large effect sizes were reported, with an unbalanced number of small studies showing a small effect, then those ‘missing’ studies are imputed, and the pooled effect size is recalculated.

Subgroup analyses were conducted to evaluate the influence of dose (i.e. <45 *v.* ≥45 g/d), study design (i.e. parallel or crossover), the control food/diet (i.e. whether it was provided or if subjects were simply instructed to avoid nuts), the duration of the study (i.e. ≥12 weeks *v.* 4 to <12 weeks (hereinafter referred to as <12 weeks)), and of baseline blood lipid level. Baseline blood lipid levels were categorised dichotomously as ‘optimal’ or ‘not optimal’, based on the targets established in the National Cholesterol Education Program Adult Treatment Panel III guidelines (i.e. optimal blood lipid levels were defined as: LDL-C < 2·59; TC < 5·17; HDL-C ≥ 1·03; TAG < 1·69 mmol/l). For crossover studies, the categorisation was based on the reported baseline lipid level; for parallel studies, the categorisation was based on the average of the baseline lipid levels that were reported for each group, weighted by the sample size of each group. Subgroup analyses were conducted when there were at least three strata available for pooling.

## Results

### Literature search results and overview of included studies

**Fig. 1.**
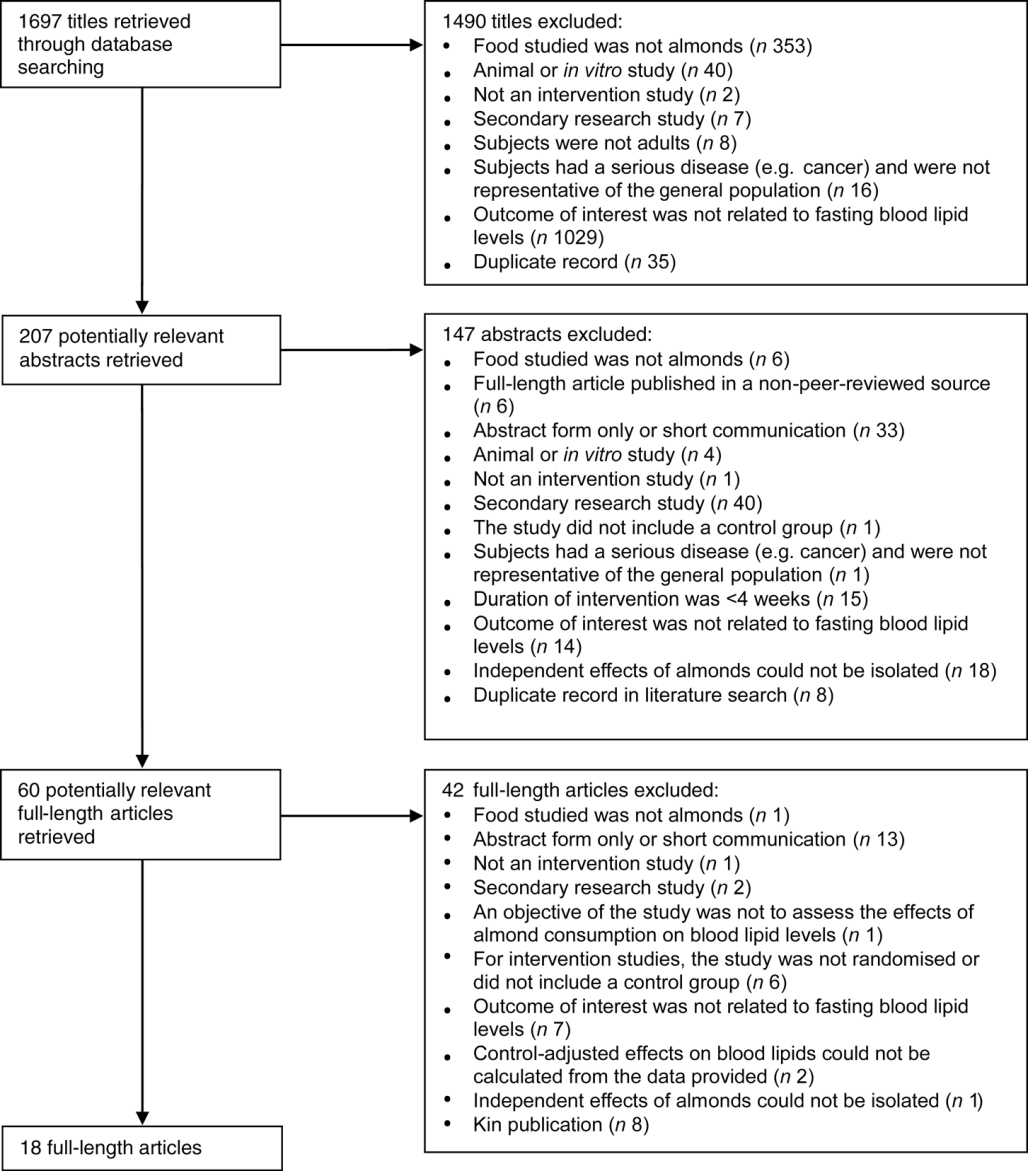
Flowchart of the literature search process.

The three literature searches resulted in the identification of 1697 titles, of which eighteen publications met all of the inclusion criteria and none of the exclusion criteria (Fig. 1).

The eighteen publications provided a total of twenty-seven strata (Table 1). Of the twenty-seven strata, seventeen were from parallel trials, and the remaining ten strata were from crossover trials. The number of study completers ranged from thirteen to 137 amongst the eighteen publications. Both male and female subjects were studied in the majority of the strata, with the exception of three strata wherein only females were studied (Abazarfard *et al.*^(^^9^^)^; Kurlandsky & Stote strata 1 and 2^(^^14^^)^) and two strata wherein only males were studied (Jia *et al.* strata 1 and 2^(^^15^^)^). The subjects were described by the authors as generally healthy in seven strata (Abazarfard *et al.*^(^^9^^)^; Berryman *et al.*^(^^11^^)^; Foster *et al.*^(^^16^^)^; Kurlandsky & Stote strata 1 and 2^(^^14^^)^; Spiller *et al.* strata 1 and 2^(^^17^^)^), generally healthy but habitual smokers in two strata (Jia *et al.* strata 1 and 2^(^^15^^)^), generally healthy or hyperlipidaemic in two strata (Sabaté *et al.* strata 1 and 2^(^^18^^)^), hyperlipidaemic in four strata (Damasceno *et al.*^(^^19^^)^; Jenkins *et al.* strata 1 and 2^(^^20^^)^; Tamizifar *et al.*^(^^21^^)^), or pre-diabetic or at risk of type 2 diabetes mellitus (T2DM) in five strata (Tan & Mattes strata 1 to 4^(^^22^^)^; Wien *et al.*^(^^23^^)^). In the remaining seven strata, medicated subjects were studied, including subjects with T2DM on oral hypoglycaemic therapy (Sweazea *et al.*^(^^10^^)^; Cohen & Johnston^(^^24^^)^; Li *et al.*^(^^25^^)^; Lovejoy *et al.* strata 1 and 2^(^^26^^)^), subjects on stable statin therapy (Ruisinger *et al.*^(^^27^^)^), and subjects on antihypertensive and/or antidiabetic medications (Wien *et al.*^(^^28^^)^).

**Table 1.** Key study characteristics of included studies (*n* 18 publications and 27 strata)

				Mean baseline				
References	Study design	Study duration	Study population (final sample size)	Age (years)	BMI (kg/m^2^)	Control	Almond intervention	Provision of control foods/diet
Abazarfard *et al.*^(^^9^^)^	R, C, P	12 weeks	100 F; generally healthy; non-medicated	42·7 ± 7·1*	29·6 ± 1·5*	No nuts (*n* 50)	50 g/d almonds (raw) as two snacks (about 25 g/snack) (*n* 50)	Hypoenergetic diet prescribed; foods were self-selected from a provided list
Berryman *et al.*^(^^11^^)^	R, C, X	6 weeks (2 weeks WO)	48 (22 M, 26 F); generally healthy; non-medicated	49·9 ± 9·4	26·2 ± 2·8	106 g/d banana muffin with 2·7 g/d butter	42·5 g/d (1·5 oz) whole almonds (unsalted, unroasted)	Meals prepared by a metabolic kitchen
Cohen & Johnston^(^^24^^)^	R, C, P	12 weeks	13 (7 M, 6 F); T2DM; medicated†	66 ± 8·4*	34·8 ± 8·0*	Two cheese sticks, 5 d/week (*n* 7)	20 g/d almonds (28 g or 1 oz, 5 d/week) (*n* 6)	Foods were self-selected
Damasceno *et al.*^(^^19^^)^‡	R, C, X, SB§	4 weeks (no WO)	18 (9 M, 9 F); hypercholesterolaemic; non-medicated	56 ± 13*	25·7 ± 2·3*	35 to 50 g/d olive oil	50 to 75 g/d almonds (raw, shelled, Spanish Marcona variety)║	Mediterranean-type diet prescribed; foods were self-selected¶
Foster *et al.*^(^^16^^)^	R, C, P	18 months	92 (M and F); generally healthy; non-medicated	46·8 ± 12·5*	34·0 ± 3·6*	No nuts or peanut butter (*n* 45)	56 g/d almonds (*n* 47)**	Low-energy diet prescribed; foods were self-selected
Jenkins *et al.* stratum 1^(^^20^^)^	R, C, X	4 weeks (≥2 weeks WO)	27 (15 M, 12 F); hyperlipidaemic; non-medicated††	64 ± 9	25·7 ± 3	147 ± 6 g/d muffin	37 ± 2 g/d whole almonds (raw, unblanched) with 75 ± 3 g/d muffin	Self-selected low-fat therapeutic diet (selected following dietary instruction)
Jenkins *et al.* stratum 2^(^^20^^)^							73 ± 3 g/d whole almonds (raw, unblanched)	
Jia *et al.* stratum 1^(^^15^^)^	R, C, P	4 weeks	30 M; generally healthy, habitual smokers; part of an army unit; medication status NR	22·3 ± 1·8*	NR	No almonds (*n* 10)	84 g/d (3 oz) almond powder (*n* 10)	Meals provided by army unit canteen
Jia *et al.* stratum 2^(^^15^^)^				22·1 ± 1·8*			168 g/d (6 oz) almond powder (*n* 10)	
Kurlandsky & Stote stratum 1^(^^14^^)^	R, C, P	6 weeks	47 F; healthy normocholesterolaemic; non-medicated‡‡	46·6 ± 9·4*	25·7 ± 3·8*	No nuts or chocolate (*n* 12)	60 g/d almonds (*n* 12)	Dark chocolate and almonds provided; foods were otherwise self-selected
Kurlandsky & Stote stratum 2^(^^14^^)^				41·1 ± 10·2*	25·5 ± 3·8*	41 g/d dark chocolate (*n* 12)	41 g/d dark chocolate and 60 g/d almonds (*n* 11)	
Li *et al.*^(^^25^^)^	R, C, X	4 weeks (2 weeks WO)	20 (9 M, 11 F); T2DM; medicated§§	58 ± 2	26·0 ± 0·7	No almonds	About 56 g/d whole almonds (roasted, unsalted, unblanched); 20 % of TDEI from almonds║║	Meals prepared by metabolic kitchen
Lovejoy *et al.* stratum 1^(^^26^^)^	R, DB, X¶¶	4 weeks (2 weeks WO)	30 (13 M, 17 F); T2DM; medicated***	53·8 ± 10·4	33·0 ± 1·0	Olive oil or canola oil; fat comprised 25 % of TDEI, with 10 % from olive or canola oil	57 to 113 g/d almonds (in meals and snacks); fat comprised 25 % of TDEI, with 10 % from almonds	Meals provided†††
Lovejoy *et al.* stratum 2^(^^26^^)^						Olive oil or canola oil; fat comprised 37 % of TDEI, with 10 % from olive or canola oil	57 to 113 g/d almonds (in meals and snacks); fat comprised 37 % of TDEI, with 10 % from almonds	
Ruisinger *et al.*^(^^27^^)^	R, C, P	4 weeks	48 (24 M, 24 F); medicated (on stable statin therapy‡‡‡)	59·6 ± 11·1*	29·2 ± 4·3*	NCEP ATP III diet counselling (*n* 26)	NCEP ATP III diet counselling and 100 g/d whole almonds (raw, unsalted) (*n* 22)	Diet counselling provided§§§
Sabaté *et al.* stratum 1^(^^18^^)^	R, C, X	4 weeks (no WO║║║)	25 (14 M, 11 F); generally healthy or mildly hypercholesterolaemic; non-medicated	NR (20 to 60)	NR¶¶¶	No almonds	About 34 g almonds/2000 kcal (in meals and snacks); 10 % of TDEI from almonds	Meals prepared by a metabolic kitchen****
Sabaté *et al.* stratum 2^(^^18^^)^							About 68 g almonds/2000 kcal (in meals and snacks); 20 % of TDEI from almonds	
Spiller *et al.* stratum 1^(^^17^^)^	R, C, P	4 weeks	45 (12 M, 33 F); generally healthy; non-medicated	53 ± 10††††	NR	48 g/d olive oil, 113 g/d cottage cheese, and 21 g/d rye crackers (*n* 15)	100 g/d whole or ground almonds (raw, unblanched) (*n* 18)	Diet was both prescribed and self-selected‡‡‡‡
Spiller *et al.* stratum 2^(^^17^^)^						85 g/d cheddar cheese, 28 g/d butter, and 21 g/d rye crackers (*n* 12)		
Sweazea *et al.*^(^^10^^)^	R, C, P	12 weeks	21 (9 M, 12 F); T2DM; medicated§§§§	56·2 ± 7·5*	35·3 ± 8·3*	No almonds (*n* 11)	43 g (1·5 oz) whole almonds, five to seven times/week (*n* 10)	Foods were self-selected
Tamizifar *et al.*^(^^21^^)^	R, SB║║║║, C, X	4 weeks (5 to 7 d WO)	30 (17 M, 13 F); hypercholesterolaemic; non-medicated	56 ± 6·1	24·1 ± 4·5	No nuts, nut butter or margarines, or nut oils	25 g/d almond powder	Foods were self-selected from a provided list
Tan & Mattes stratum 1^(^^22^^)^	R, C, P	4 weeks	137 (48 M, 89 F); at risk of T2DM; non-medicated¶¶¶¶	30·8 ± 10·6*	27·6 ± 4·6*	No nuts or seeds (*n* 27)	43 g/d almonds with breakfast (*n* 28)	Foods were self-selected
Tan & Mattes stratum 2^(^^22^^)^				28·2 ± 10·2*	27·9 ± 4·7*		43 g/d almonds as morning snack (2 h after breakfast and 2 h before lunch) (*n* 28)	
Tan & Mattes stratum 3^(^^22^^)^				29·0 ± 11·7*	28·0 ± 4·2*		43 g/d almonds with lunch (*n* 26)	
Tan & Mattes stratum 4^(^^22^^)^				28·9 ± 10·8*	27·6 ± 4·8*		43 g/d almonds as afternoon snack (2 h after lunch and 2 h before dinner) (*n* 28)	
Wien *et al.*^(^^28^^)^	R, C, P	24 weeks	52 (M and F); with a medical diagnosis that could benefit from weight reduction*****	55 ± 2·0*	38·0 ± 1·0*	Low-energy liquid formula + complex CHO (*n* 28)	Low-energy liquid formula + 84 g/d whole, unblanched almonds (*n* 24)	Low-energy liquid formula provided; complex CHO foods were self-selected from a provided list
Wien *et al.*^(^^23^^)^	R, C, P	16 weeks	54 (M and F); prediabetic†††††; non-medicated‡‡‡‡‡	53·5 ± 10·1*	29·5 ± 5*	No tree nuts or peanuts (*n* 29)	About 60 g/d almonds (raw or dry roasted); 20 % of TDEI from almonds (*n* 25)	Foods were self-selected

R, randomised; C, controlled; P, parallel; F, females; X, crossover; WO, washout; M, males; T2DM, type 2 diabetes mellitus; SB, single-blinded; NR, not reported; TDEI, total daily energy intake; DB, double-blinded; NCEP ATP III, National Cholesterol Education Program Adult Treatment Panel's Third Report on Therapeutic Lifestyle Changes; CHO, carbohydrates; ACE, angiotensin-converting enzyme; HRT, hormone replacement therapy; ADA, American Diabetes Association.

* For P studies, the weighted mean age/BMI and pooled sd were calculated by using the mean age/BMI and sd values that were reported separately for the individual groups.

† Subjects had not been prescribed insulin; rather, subjects were on stable oral hypoglycaemic therapy.

‡ In another arm of this trial, subjects were administered 40 to 65 g/d of walnuts; however, according to the study exclusion criteria, the control is not acceptable if it consists of another tree nut or any tree nut fraction. Thus, the results of the walnuts intervention arm were excluded from the analysis.

§ Damasceno *et al.*^(^^19^^)^ acknowledged that, given the nature of the foods provided, which could not be masked, the study was unblinded; however, it was noted that investigators involved in the preparation of databases and laboratory determinations were masked with respect to treatment sequence.

║ The almonds partially replaced other MUFA-rich foods, such as olives and avocados.

¶ The Mediterranean-type, cholesterol-lowering diet was composed of natural foodstuffs. Vegetable products and fish were emphasised, while red and processed meats, whole-fat dairy products and eggs were limited. Recipes using nuts were provided to the subjects who consumed the nuts with meals in desserts or salads or as snacks.

** During the first 5 weeks, subjects received whole, raw almonds only. From week 6, roasted almonds were introduced and, over time, a variety of isoenergetic, flavoured almonds were used.

†† The majority of the subjects were not medicated; however, 3 M and 5 F were taking the following medications: statins (*n* 2), β-blocking agents (*n* 3), ACE inhibitors (*n* 3), angiotensin II AT1 receptor blockers (*n* 1), thiazide diuretics (*n* 2), levothyroxine (*n* 2) and HRT (*n* 2). Medication dosages were held constant throughout the study.

‡‡ Subjects did not use lipid-lowering medications or dietary supplements. Subjects were allowed to be on stable regimens of oral contraceptives and HRT.

§§ Subjects did not receive insulin therapy; rather, all subjects were on stable oral hypoglycaemic therapy.

║║ Almonds were incorporated into the control diet to replace 20 % of TDEI in the control diet; depending on the menus, almonds were either incorporated into entrées and desserts or consumed as a snack.

¶¶ In this study, subjects were provided with all foods needed for the duration of the study; since the study was crossover in design but described as DB, it is assumed that almond powder was used in the formulation of the entrées and snacks. However, it remains unclear how true double-blinding was achieved, given that the subjects could have tasted the almonds in their foods (it is possible that the ‘control’ foods were flavoured with an almond extract; however, this was not described in the publication).

*** Subjects on lipid-lowering or insulin therapy were excluded. Eleven F received HRT, and sixteen of the subjects were taking oral hypoglycaemic agents.

††† Regarding background diets, on weekdays, the subjects were required to consume breakfast and dinner under supervision at the Pennington Biomedical Research Center's dining facility; weekday lunches and snacks and all weekend meals were packaged for take-out.

‡‡‡ Subjects were taking chronic statin therapy, defined as a consistent statin dose for at least 8 weeks before study entry with continuation of the same dose during the 4-week study period. Subjects who took lipid-lowering agents other than statins were excluded. Post-menopausal F who were not taking HRT or were on a consistent HRT dose were included. F of child-bearing potential using an effective form of contraception were allowed to participate in the study.

§§§ Subjects received NCEP ATP III diet counselling via telephone and instructions on how to compensate for the added energy from the almonds.

║║║ The authors argued that a WO was not included because serum lipids and lipoproteins are known to stabilise within 3 weeks.

¶¶¶ Although the mean BMI was NR, it should be noted that a BMI >30 kg/m^2^ was an exclusion criterion.

**** On Sunday to Friday of each week, subjects ate breakfast and dinner at the Loma Linda University metabolic kitchen. Lunch meals and all Saturday meals were packaged for consumption away from the metabolic kitchen.

†††† The mean age was provided for the forty-five study completers. Because the mean age was not provided for each group, the weighted mean age for each comparison could not be calculated.

‡‡‡‡ All subjects were provided with whole-grain bread, brown rice, pasta, non-fat yogurt, rice cakes, dry beans, lentils, and couscous and were instructed to eat these foods a set number of times during each week. Subjects rounded out their daily food intake with fruits, vegetables, other whole grains, legumes, low-fat or non-fat milk (whole milk dairy products were not allowed), egg whites and lean fish. Lean beef was allowed twice weekly, and poultry and fatty fish were permitted up to four times weekly. Up to four whole eggs were permitted/week, but only if the subject had been consuming eggs prior to the study. Foods not allowed included: commercial or homemade products containing fats other than the study fat, and products made with refined flour (e.g. snack foods, chips, crackers, cakes, pastries, pies, candy or ice cream). Usual coffee, tea, alcohol and soft drink consumption was permitted.

§§§§ Subjects on insulin therapy were excluded. Subjects taking prescription medications, including oral hypoglycaemic agents, statins or hypertensive medications were instructed to maintain consistent use throughout the study.

║║║║ The study by Tamizifar *et al.*^(^^21^^)^ was described as SB. Given that, during the almond phase of the crossover study, the subjects were given almond powder and, during the control phase, the subjects were not administered anything, it is assumed that the investigators were blinded, and not the subjects.

¶¶¶¶ Subjects were considered at risk of T2DM if they were overweight or obese (BMI >27 kg/m^2^) or were normal weight (BMI 18·5–24·9 kg/m^2^) but had a strong family history for T2DM.

***** The proportion of subjects with a diagnosis of hypertension at baseline was 62 % in the control group and 50 % in the almond group; the proportion of subjects with T2DM was NR. However, it was noted that during randomisation, subjects were stratified according to the presence or absence of T2DM.

††††† Prediabetes was diagnosed according to the 2005 ADA diagnostic guidelines: fasting blood glucose between 100 and 125 mg/dl (5·56 and 6·94 mmol/l) or casual blood glucose ≥140–199 mg/dl (≥7·78–11·06 mmol/l).

‡‡‡‡‡ Subjects taking corticosteroids or immunosuppressant medications were excluded. Two subjects in each group were taking lipid-lowering medications.

### Almond interventions

Across all strata, the average daily intake of almonds ranged from 20 to 113 g/d, and the duration of the almond consumption period ranged from 4 weeks to 18 months. Almonds were required to be consumed every day in all studies except two, in which 28 g (1 oz) of almonds were required to be consumed 5 d per week^(^^24^^)^ or 43 g (1·5 oz) of almonds were required to be consumed five to seven times weekly^(^^10^^)^.

Whole, raw (unblanched, unsalted) almonds were consumed in nine strata (Abazarfard *et al.*^(^^9^^)^; Sweazea *et al.*^(^^10^^)^; Damasceno *et al.*^(^^19^^)^; Jenkins *et al.* strata 1 and 2^(^^20^^)^; Ruisinger *et al.*^(^^27^^)^; Spiller *et al.* strata 1 and 2^(^^17^^)^; Wien *et al.*^(^^28^^)^). In five strata (Cohen & Johnston^(^^24^^)^; Tan & Mattes strata 1 to 4^(^^22^^)^), the almonds that were consumed by the subjects were not specifically described by the study authors as raw, unblanched almonds; however, based on the reported energy value of the almonds, it was determined that the almonds were most probably raw, unblanched almonds. A variety of almonds, namely whole (raw), roasted, and flavoured almonds were consumed in one stratum (Foster *et al.*^(^^16^^)^); dry, roasted almonds were consumed in one stratum (Wien *et al.*^(^^23^^)^); and almond powder was consumed in one stratum (Tamizifar *et al.*^(^^21^^)^). The types of almonds used were not specified in two strata (Kurlandsky & Stote strata 1 and 2^(^^14^^)^). In eight strata, all meals and snacks were provided and the almonds were said to have been consumed as a snack (Berryman *et al.*^(^^11^) or incorporated into the meals and snacks (Jia *et al.* strata 1 and 2^(^^15^^)^; Li *et al.*^(^^25^^)^; Lovejoy *et al.* strata 1 and 2^(^^26^^)^; Sabaté *et al.* strata 1 and 2^(^^18^^)^). The form of almonds that was used was described only by Berryman *et al.*^(^^11^^)^, who reported administering unsalted, whole, natural almonds with skins, and by Jia *et al.* strata 1 and 2^(^^15^^)^, who reported using almond powder. In the remaining five strata in which all meals and snacks were provided (Li *et al.*^(^^25^^)^; Lovejoy *et al.* strata 1 and 2^(^^26^^)^; Sabaté *et al.* strata 1 and 2^(^^18^^)^), it is assumed that whole almonds, almond pieces and ground almonds were used to prepare the meals.

### Control foods/diets

Although all studies were randomised and controlled, the control food was not defined in some studies but defined in other studies. In thirteen of the twenty-seven strata, subjects in the control group or during the control phase were instructed not to consume nuts, but were not provided with a control food or with a control diet (Abazarfard *et al.*^(^^9^^)^; Sweazea *et al.*^(^^10^^)^; Foster *et al.*^(^^16^^)^; Kurlandsky & Stote strata 1 and 2^(^^14^^)^; Ruisinger *et al.*^(^^27^^)^; Tamizifar *et al.*^(^^21^^)^; Tan & Mattes strata 1 to 4^(^^22^^)^; Wien *et al.*^(^^28^^)^; Wien *et al.*^(^^23^^)^). In fourteen strata, either a ‘control food’ (e.g. cheese sticks or a muffin or olive oil) was provided (Cohen & Johnston^(^^24^^)^; Damasceno *et al.* ^(^^19^^)^; Jenkins *et al.* strata 1 and 2^(^^20^^)^; Spiller *et al.* strata 1 and 2^(^^17^^)^), or the entire control diet was provided (Berryman *et al.*^(^^11^^)^; Jia *et al.* strata 1 and 2^(^^15^^)^; Li *et al.*^(^^25^^)^; Lovejoy *et al.* strata 1 and 2^(^^26^^)^; Sabaté *et al.* strata 1 and 2^(^^18^^)^).

### Study quality

Based on Health Canada's quality appraisal tool, all of the studies were considered to be ‘higher quality’^(^^8^^)^. Across all eighteen publications, the most commonly identified limitations included the lack of reporting on allocation concealment (*n* 16), the lack of reporting on the method of randomisation and thus the ‘appropriateness’ of the randomisation method, which also is a quality factor, could not be determined (*n* 13), as well as the lack of reporting of an intent-to-treat analysis (*n* 13).

### Effects of almonds on fasting blood lipids

The fasting blood lipids that were assessed in each of the twenty-seven strata, as well as other information pertinent to the subgroup analyses (i.e. study design, almond intake, whether a control food/diet was provided, study duration, and baseline blood lipid levels), are summarised in Table 2. In the study by Lovejoy *et al.* strata 1 and 2^(^^26^^)^, baseline TAG levels were not reported; thus, a determination as to whether TAG levels at baseline were or were not optimal could not be made. In the study by Spiller *et al.* strata 1 and 2^(^^17^^)^, TC, LDL-C, HDL-C and TAG were assessed at baseline and at the end of treatment; however, only the results for TC and LDL-C at the end of treatment were reported. Thus, the HDL-C and TAG results could not be included in the meta-analyses, and in order to include the results related to TC and LDL-C in the meta-analyses, the mean difference in the effect for TC and LDL-C had to be calculated by subtracting the end-of-treatment value in the control group from the end-of-treatment value in the almond group (as opposed to subtracting the changes from baseline in the control group from the changes from baseline in the almond group, which was done for all other parallel studies).

**Table 2.** Summary of study design and duration, almond dose and baseline blood lipids

References	Study design		Dose (g/d)		Control food/diet		Duration (weeks)		TC	LDL-C			HDL-C	TAG			TC: HDL-C	LDL-C: HDL-C
*P*	X	≥45	<45	Pr	NPr	<12	≥12	BL*	EOT	BL*	EOT	BL*	EOT	BL*	EOT
Abazarfard *et al.*^(^^9^^)^	✓		✓			✓		✓	N.O.	✓	N.O.	✓	O	✓	N.O.	✓	✓	–
Berryman *et al.*^(^^11^^)^		✓		✓	✓		✓		N.O.	✓	N.O.	✓	O	✓	O	✓	✓	✓
Cohen & Johnston^(^^24^^)^	✓			✓	✓			✓	O	✓	O	✓	–	–	N.O.	✓	–	–
Damasceno *et al.*^(^^19^^)^		✓	✓		✓		✓		N.O.	✓	N.O.	✓	O	✓	O	✓	–	✓
Foster *et al.*^(^^16^^)^	✓		✓			✓		✓	O	✓	N.O.	✓	O	✓	O	✓	✓	–
Jenkins *et al.* stratum 1^(^^20^^)^		✓		✓	✓		✓		N.O.	✓	N.O.	✓	O	✓	N.O.	✓	✓	✓
Jenkins *et al.* stratum 2^(^^20^^)^		✓	✓		✓		✓		N.O.	✓	N.O.	✓	O	✓	O	✓	✓	✓
Jia *et al.* stratum 1^(^^15^^)^	✓		✓		✓		✓		O	✓	–	–	–	–	O	✓	–	–
Jia *et al.* stratum 2^(^^15^^)^	✓		✓		✓		✓		O	✓	–	–	–	–	O	✓	–	–
Kurlandsky & Stote stratum 1^(^^14^^)^	✓		✓			✓	✓		N.O.	✓	N.O.	✓	O	✓	O	✓	–	–
Kurlandsky & Stote stratum 2^(^^14^^)^	✓		✓			✓	✓		O	✓	N.O.	✓	O	✓	O	✓	–	–
Li *et al.*^(^^25^^)^		✓	✓		✓		✓		N.O.	✓	N.O.	✓	O	✓	O	✓	–	✓
Lovejoy *et al.* stratum 1^(^^26^^)^		✓	✓		✓		✓		O	✓	N.O.	✓	O	✓	–	✓	✓	✓
Lovejoy *et al.* stratum 2^(^^26^^)^		✓	✓		✓		✓		O	✓	N.O.	✓	O	✓	–	✓	✓	✓
Ruisinger *et al.*^(^^27^^)^	✓		✓			✓	✓		O	✓	N.O.	✓	O	✓	N.O.	✓	–	–
Sabaté *et al.* stratum 1^(^^18^^)^		✓		✓	✓		✓		N.O.	✓	N.O.	✓	O	✓	O	✓	–	✓
Sabaté *et al.* stratum 2^(^^18^^)^		✓	✓		✓		✓		N.O.	✓	N.O.	✓	O	✓	O	✓	–	✓
Spiller *et al.* stratum 1^(^^17^^)^	✓		✓		✓		✓		N.O.	✓	N.O.	✓	–†	–†	–†	–†	–	–
Spiller *et al.* stratum 2^(^^17^^)^	✓		✓		✓		✓		N.O.	✓	N.O.	✓	–†	–†	–†	–†	–	–
Sweazea *et al.*^(^^10^^)^	✓			✓		✓		✓	O	✓	N.O.	✓	O	✓	N.O.	✓	–	–
Tamizifar *et al.*^(^^21^^)^		✓		✓		✓	✓		N.O.	✓	N.O.	✓	N.O.	✓	N.O.	✓	✓	–
Tan & Mattes stratum 1^(^^22^^)^	✓			✓		✓	✓		O	✓	O	✓	O	✓	O	✓	–	–
Tan & Mattes stratum 2^(^^22^^)^	✓			✓		✓	✓		O	✓	O	✓	O	✓	O	✓	–	–
Tan & Mattes stratum 3^(^^22^^)^	✓			✓		✓	✓		O	✓	O	✓	O	✓	O	✓	–	–
Tan & Mattes stratum 4^(^^22^^)^	✓			✓		✓	✓		O	✓	O	✓	O	✓	O	✓	–	–
Wien *et al.*^(^^28^^)^	✓		✓			✓		✓	N.O.	✓	N.O.	✓	N.O.	✓	N.O.	✓	–	✓
Wien *et al.*^(^^23^^)^	✓		✓			✓		✓	N.O.	✓	N.O.	✓	O	✓	O	✓	✓	–
Total	17	10	17	10	14	13	21	6	14 N.O. 13 O	27	20 N.O. 5 O	25	2 N.O. 20 O	22	7 N.O. 16 O	25	9	10

TC, total cholesterol; LDL-C; LDL-cholesterol; HDL-C; HDL-cholesterol; P, parallel; X, crossover; Pr, provided; NPr, not provided; BL, baseline; EOT, end of treatment; N.O., not optimal; O, optimal; –, not reported.

* Mean baseline TC, LDL-C, HDL-C and TAG were categorised as O or N.O., based on the targets established in the National Cholesterol Education Program Adult Treatment Panel III guidelines (i.e. optimal blood lipid levels were defined as: TC < 5·17 mmol/l; LDL-C < 2·59 mmol/l; HDL-C ≥ 1·03 mmol/l; TAG < ·69 mmol/l).

† In the study by Spiller *et al.* strata 1 and 2^(^^17^^)^, HDL-C and TAG levels were assessed at BL and at EOT; however, values were presented only in figure form, with no measures of variability. The results related to HDL-C and TAG could not be included in the meta-analyses.

**Fig. 2.**
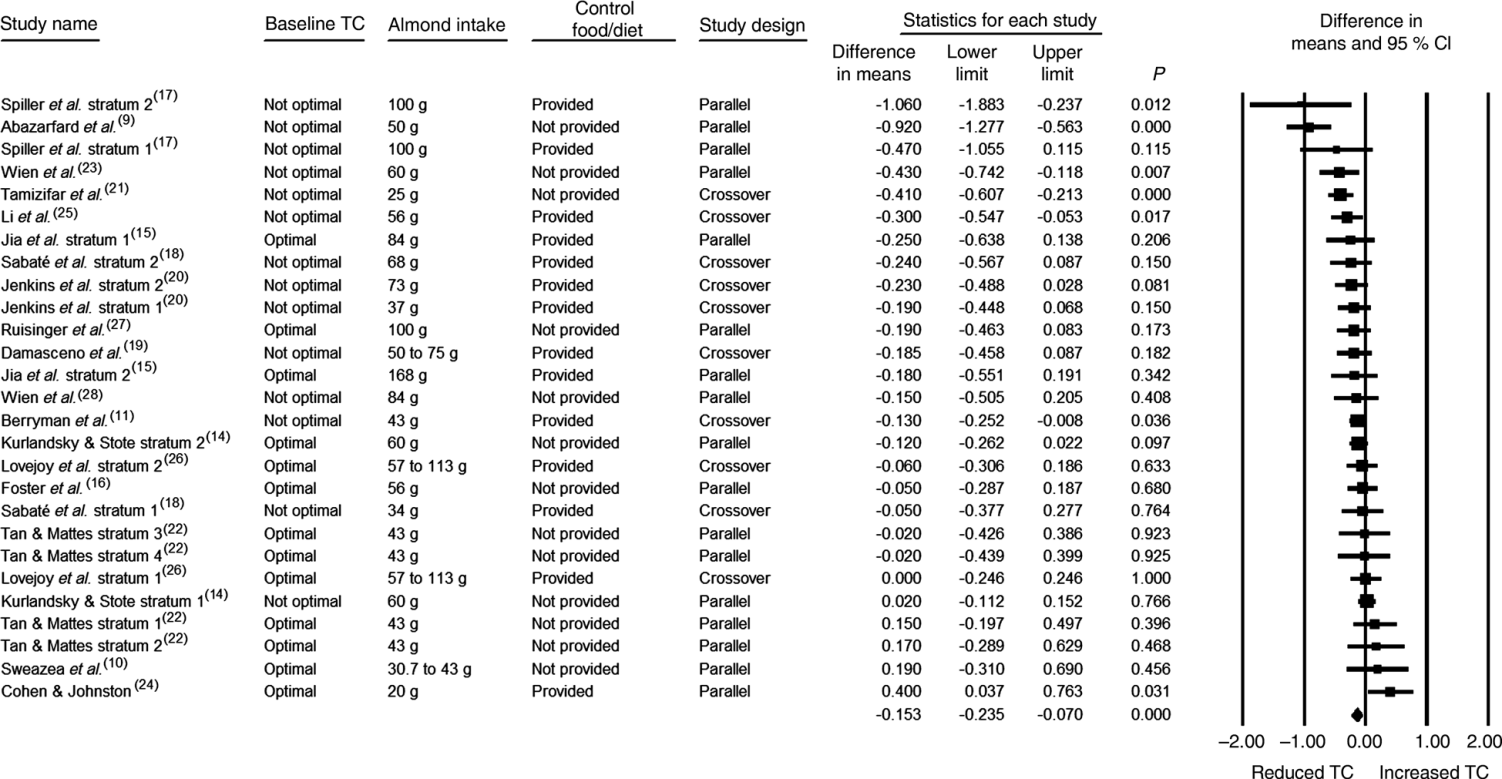
Effect of almond consumption on total cholesterol (TC).

The effects of almonds on fasting TC levels were assessed in all twenty-seven strata. The daily almond intake was 45 g or greater in 63 % of the strata, the design was crossover in 37 % of the strata, the baseline fasting TC level was not optimal in 52 % of the strata, a control food/diet was provided in 52 % of the strata, and the study duration was <12 weeks in 78 % of the strata (Table 3). As can be seen in Table 3 and Fig. 2, the reduction in TC was statistically significant when data from all twenty-seven strata were pooled (−0·153 mmol/l; 95 % CI −0·235, −0·070 mmol/l; *P* < 0·001). As there was no publication bias identified, no adjustment to these values was made. In all of the subgroup analyses, the pooled effect sizes for TC were negative. Statistical significance was observed when pooling those strata in which the almond dose was ≥45 g/d, the baseline TC level was not optimal, the study design was either parallel or crossover, the control food/diet either was or was not provided, and the duration of the almond intervention period was <12 weeks (Table 3).

**Table 3.** Effects of almonds on blood lipid levels: results of meta-analyses of randomised controlled trials*†

		TC (mmol/l)	LDL-C (mmol/l)	HDL-C (mmol/l)	TAG (mmol/l)	TC:HDL-C	LDL-C:HDL-C
All strata		*n* 27	*n* 25	*n* 22	*n* 25	*n* 9	*n* 10
		−0·153 (−0·235, −0·070)	−0·124 (−0·196, −0·051)	−0·017 (−0·043, 0·009)	−0·067 (−0·132, −0·002)	−0·207 (−0·362, −0·052)	−0·089 (−0·209, 0·031)
		*P* < 0·001	*P* = 0·001	*P* = 0·207	*P* = 0·042	*P* = 0·009	*P* = 0·145
Almond dose (g/d)	≥45	*n* 17	*n* 15	*n* 13	*n* 15	*n* 6	*n* 7
		−0·212 (−0·315, −0·108)	−0·132 (−0·209, −0·054)	−0·020 (−0·050, 0·010)	−0·071 (−0·159, 0·017)	−0·173 (−0·382, 0·037)	−0·065 (−0·230, 0·101)
		*P* < 0·001	*P* = 0·001	*P* = 0·188	*P* = 0·114	*P* = 0·106	*P* = 0·445
	<45	*n* 10	*n* 10	*n* 9	*n* 10	*n* 3	*n* 3
		−0·039 (−0·188, 0·109)	−0·060 (−0·223, 0·103)	−0·008 (−0·071, 0·055)	−0·064 (−0·162, 0·034)	−0·260 (−0·404, −0·116)	−0·186 (−0·279, −0·094)
		*P* = 0·605	*P* = 0·470	*P* = 0·808	*P* = 0·199	*P* < 0·001	*P* < 0·001
Baseline lipid level	Not optimal	*n* 14	*n* 20	*n* 2; NA	*n* 7	NA	NA
		−0·271 (−0·394, −0·148)	−0·158 (−0·238, −0·078)		−0·189 (−0·447, 0·069)		
		*P* < 0·001	*P* < 0·001		*P* = 0·151		
	Optimal	*n* 13	*n* 5	*n* 20	*n* 16	NA	NA
		−0·044 (−0·125, 0·038)	0·100 (−0·064, 0·265)	0·003 (−0·016, 0·021)	−0·034 (−0·075, 0·007)		
		*P* = 0·294	*P* = 0·232	*P* = 0·773	*P* = 0·100		
Study design	Crossover	*n* 10	*n* 10	*n* 10	*n* 10	*n* 6	*n* 9
		−0·182 (−0·261, −0·103)	−0·205 (−0·316, −0·094)	−0·017 (−0·066, 0·031)	−0·017 (−0·112, 0·077)	−0·147 (−0·320, 0·026)	−0·138 (−0·210, −0·066)
		*P* < 0·001	*P* < 0·001	*P* = 0·485	*P* = 0·723	*P* = 0·096	*P* < 0·001
	Parallel	*n* 17	*n* 15	*n* 12	*n* 15	*n* 3	*n* 1; NA
		−0·135 (−0·268, −0·002)	−0·048 (−0·117, 0·022)	−0·014 (−0·052, 0·023)	−0·111 (−0·204, −0·017)	−0·336 (−0·693, 0·021)	
		*P* = 0·047	*P* = 0·178	*P* = 0·456	*P* = 0·020	*P* = 0·065	
Control food/diet	Provided	*n* 14	*n* 12	*n* 9	*n* 12	*n* 5	*n* 9
		−0·147 (−0·241, −0·053)	−0·155 (−0·241, −0·069)	0·015 (−0·008, 0·038)	−0·062 (−0·132, 0·008)	−0·100 (−0·266, 0·067)	−0·138 (−0·210, −0·066)
		*P* = 0·002	*P* < 0·001	*P* = 0·189	*P* = 0·081	*P* = 0·241	*P* < 0·001
	Not provided	*n* 13	*n* 13	*n* 13	*n* 13	*n* 3	*n* 1; NA
		−0·152 (−0·293, −0·011)	−0·093 (−0·193, 0·007)	−0·028 (−0·069, 0·014)	−0·085 (−0·188, 0·018)	−0·386 (−0·688, −0·084)	
		*P* = 0·034	*P* = 0·068	*P* = 0·188	*P* = 0·106	*P* = 0·012	
Duration (weeks)	<12	*n* 21	*n* 19	*n* 19	*n* 19	*n* 6	*n* 9
		−0·141 (−0·210, −0·072)	−0·151 (−0·231, −0·071)	−0·013 (−0·048, 0·022)	−0·046 (−0·100, 0·009)	−0·147 (−0·320, 0·026)	−0·138 (−0·210, −0·066)
		*P* < 0·001	*P* < 0·001	*P* = 0·468	*P* = 0·101	*P* = 0·096	*P* < 0·001
	≥12	*n* 6	*n* 6	*n* 5	*n* 6	*n* 3	*n* 1; NA
		−0·169 (−0·520, 0·182)	−0·031 (−0·142, 0·079)	−0·027 (−0·100, 0·046)	−0·155 (−0·416, 0·106)	−0·336 (−0·693, 0·021)	
		*P* = 0·346	*P* = 0·576	*P* = 0·467	*P* = 0·245	*P* = 0·065	

TC, total cholesterol; LDL-C, LDL-cholesterol; HDL-C, HDL-cholesterol; NA, not applicable.

* For each meta-analysis, the results indicate (in vertical order from top to bottom): *n* (the number of strata), the pooled effect/point estimate, the 95 % CI, and the statistical significance of the point estimate. Subgroup analyses were conducted if there were at least three or more strata.

† An assessment of publication bias was conducted for each lipid parameter, but only for the meta-analysis that included all strata. Publication bias was not identified for TC, LDL-C, HDL-C or TAG. For the ratio of TC:HDL-C, two studies were found to be missing to the right of the pooled effect, and with the missing studies imputed, the pooled effect was −0·120 (95 % CI −0·289, 0·050). For the ratio of LDL-C:HDL-C, two studies were found to be missing to the right of the pooled effect, and with the missing studies imputed, the pooled effect was −0·059 (95 % CI −0·175, 0·056).

**Fig. 3.**
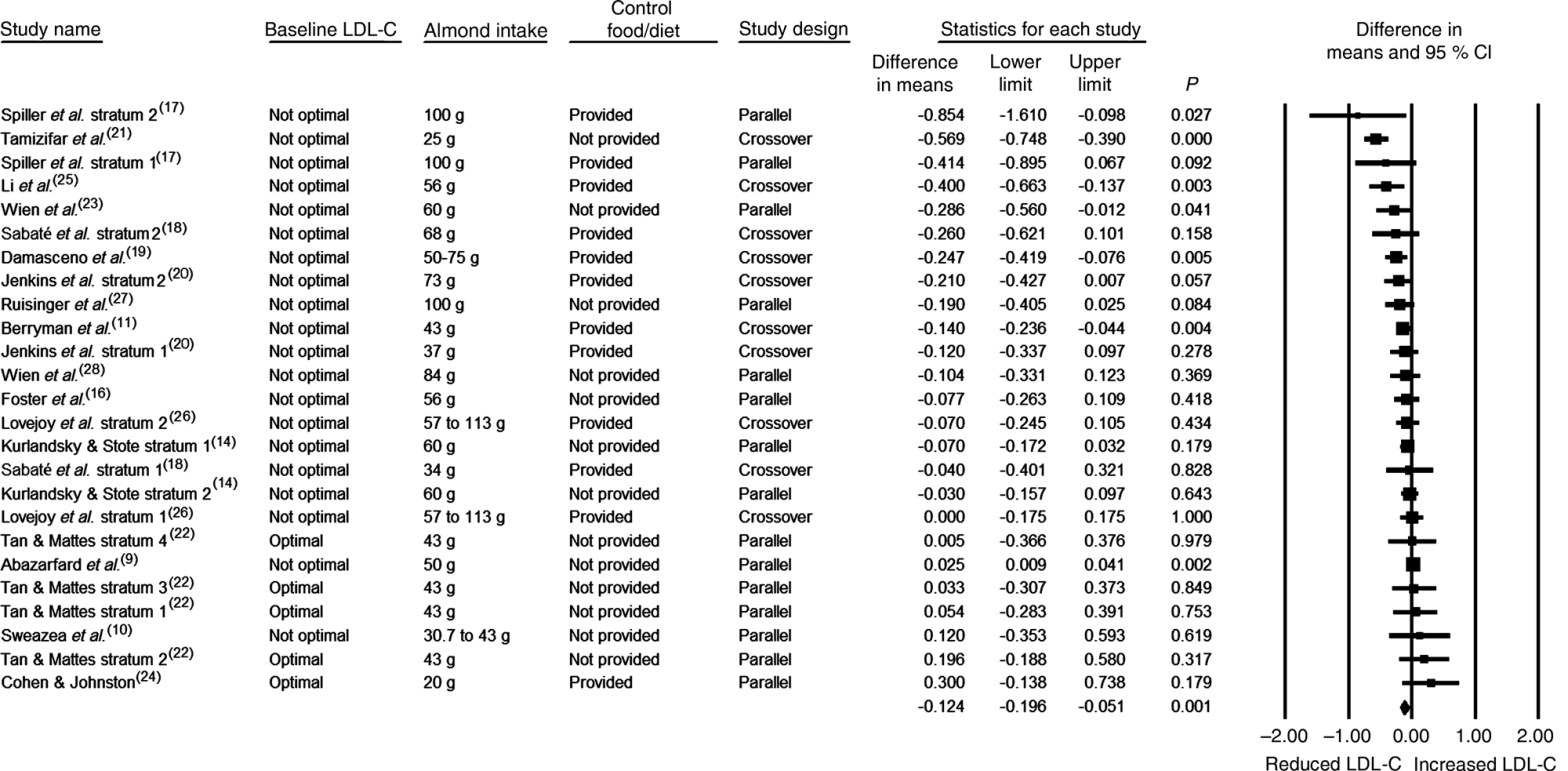
Effect of almond consumption on LDL-cholesterol (LDL-C).

The effects of almonds on fasting LDL-C were assessed in twenty-five strata. The daily almond intake was 45 g or greater in 60 % of the strata, the design was crossover in 40 % of the strata, the baseline fasting LDL-C level was not optimal in 80 % of the strata, a control food/diet was provided in 48 % of the strata, and the study duration was <12 weeks in 76 % of the strata (Table 3). As can be seen in Table 3 and Fig. 3, the reduction in LDL-C was statistically significant when data from all twenty-five strata were pooled (−0·124 mmol/l; 95 % CI −0·196, −0·051 mmol/l; *P* = 0·001). As there was no publication bias identified, no adjustment to these values was made. In all of the subgroup analyses, the pooled effect sizes for LDL-C were negative, except for when the five strata in which the baseline blood lipid level was optimal were pooled. For the subgroup analyses, statistically significant pooled reductions in LDL-C were observed when pooling those strata in which the almond dose was ≥45 g/d, the baseline LDL-C level was not optimal, the study design was crossover, a control food/diet was provided, and the study duration was <12 weeks (Table 3). It should be noted that when the control-adjusted changes in LDL-C for the thirteen strata in which the control food/diet was not provided were pooled, the reduction in LDL-C approached statistical significance (*P* = 0·068).

**Fig. 4.**
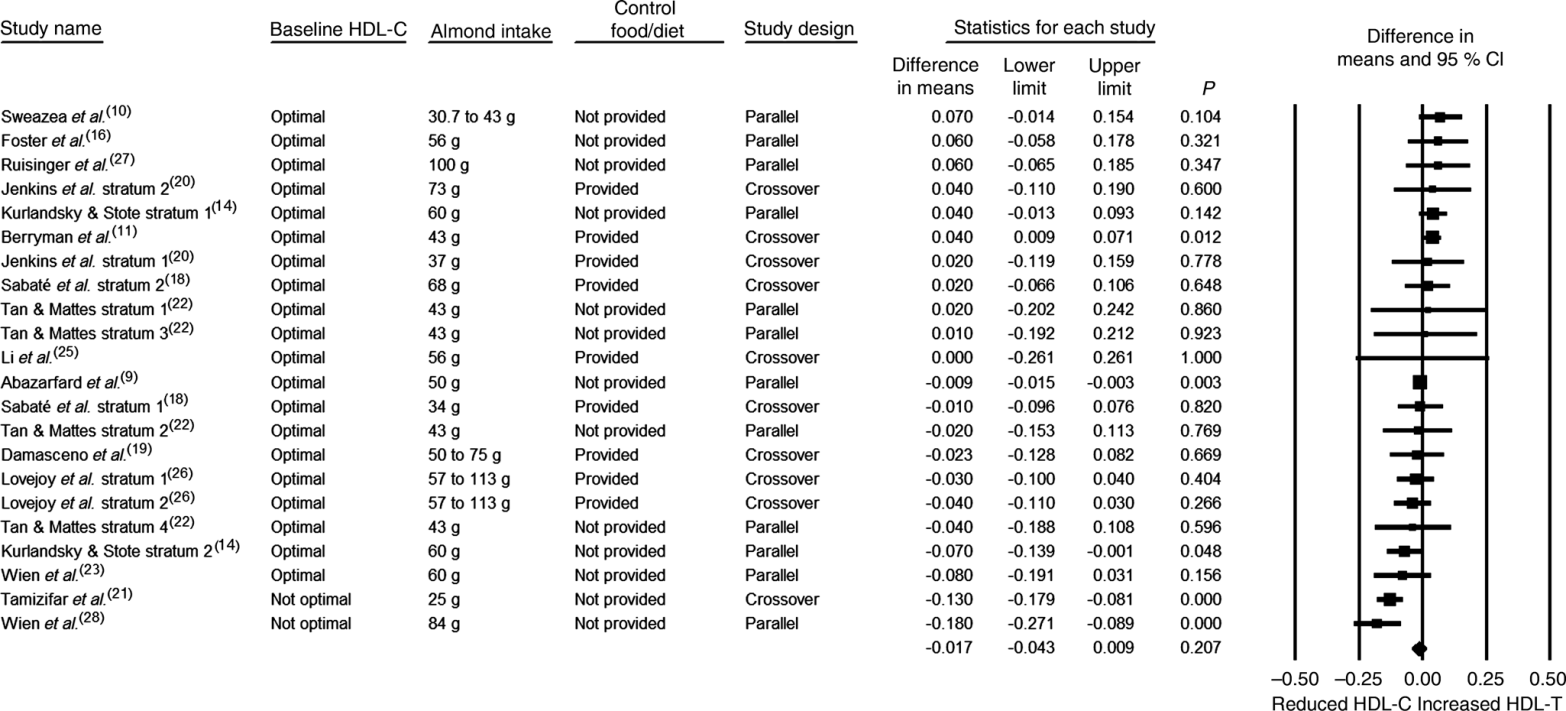
Effect of almond consumption on HDL-cholesterol (HDL-C).

Almond consumption was not associated with any significant effect on fasting HDL-C, either in the overall analysis in which all twenty-two strata were pooled or in any of the subgroup analyses (Table 3 and Fig. 4).

**Fig. 5.**
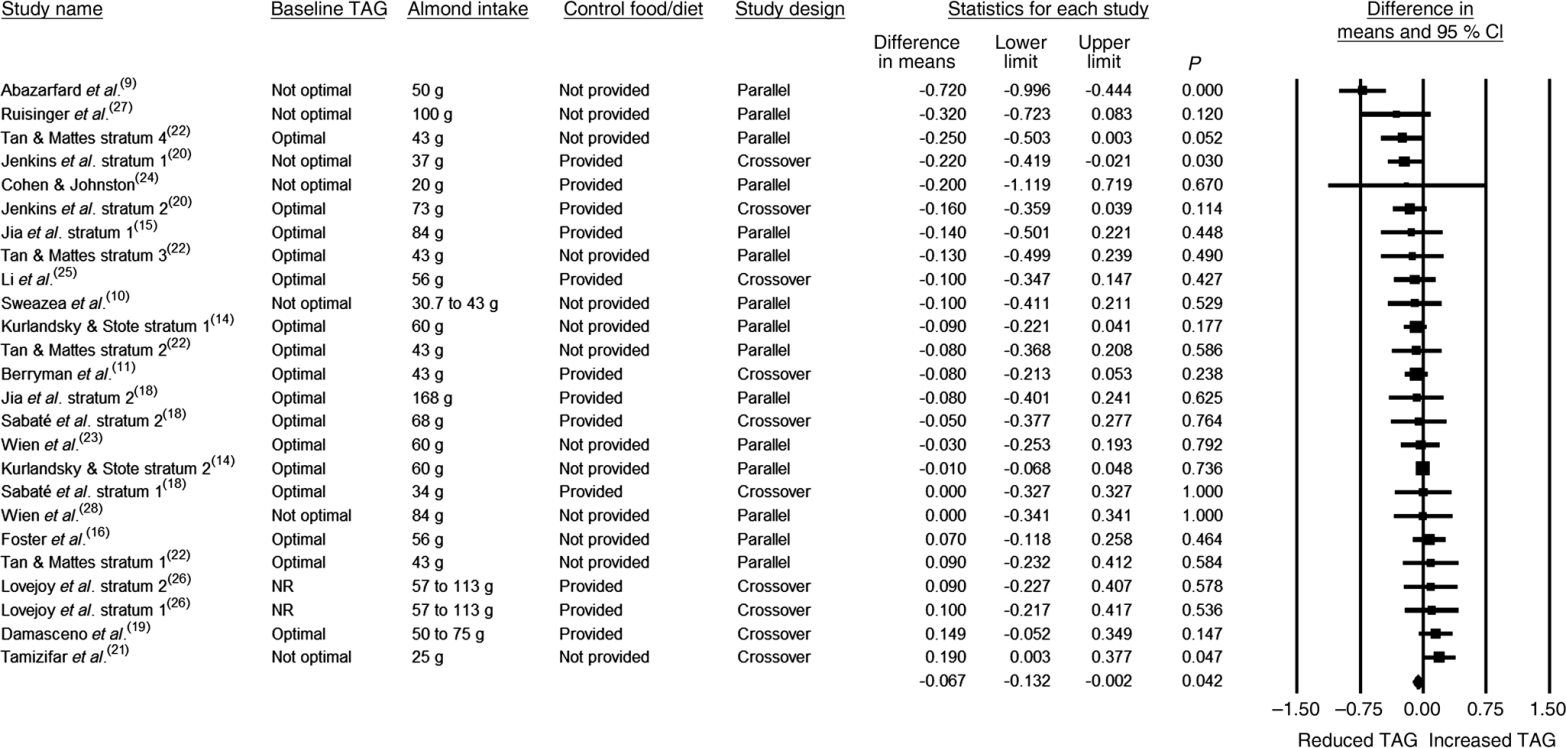
Effect of almond consumption on TAG.

The effects of almonds on fasting TAG were assessed in twenty-five strata. The almond intake was 45 g or greater in 60 % of the strata, the design was crossover in 40 % of the strata, the baseline fasting TAG level was not optimal in 30 % of the strata, a control food/diet was provided in 48 % of the strata, and the study duration was <12 weeks in 76 % of the strata (Table 3). As can be seen in Table 3 and Fig. 5, the reduction in TAG was statistically significant when data from all twenty-five strata were pooled (−0·067 mmol/l; 95 % CI −0·132, −0·002 mmol/l; *P* = 0·042). As there was no publication bias identified, no adjustment to these values was made. In all of the subgroup analyses, the pooled effect sizes for TAG were negative but not statistically significant, except for when the fifteen strata in which the study design was parallel were pooled, and the resultant pooled effect was a statistically significant reduction in TAG (−0·111 mmol/l; 95 % CI −0·204, −0·017; *P* = 0·020). Through additional sensitivity analyses, it was determined that this effect was dependent on the inclusion of the parallel study by Abazarfard *et al.*^(^^9^^)^, which included 100 females and was a relatively larger study.

**Fig. 6.**
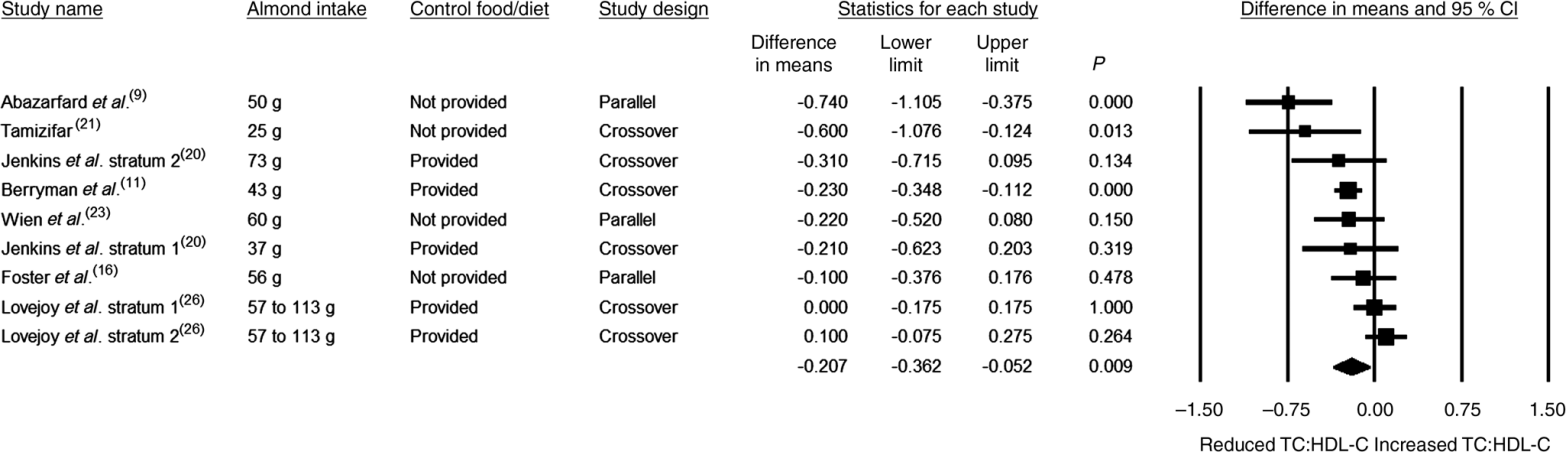
Effect of almond consumption on the ratio of total cholesterol:HDL-cholesterol (TC:HDL-C).

**Fig. 7.**
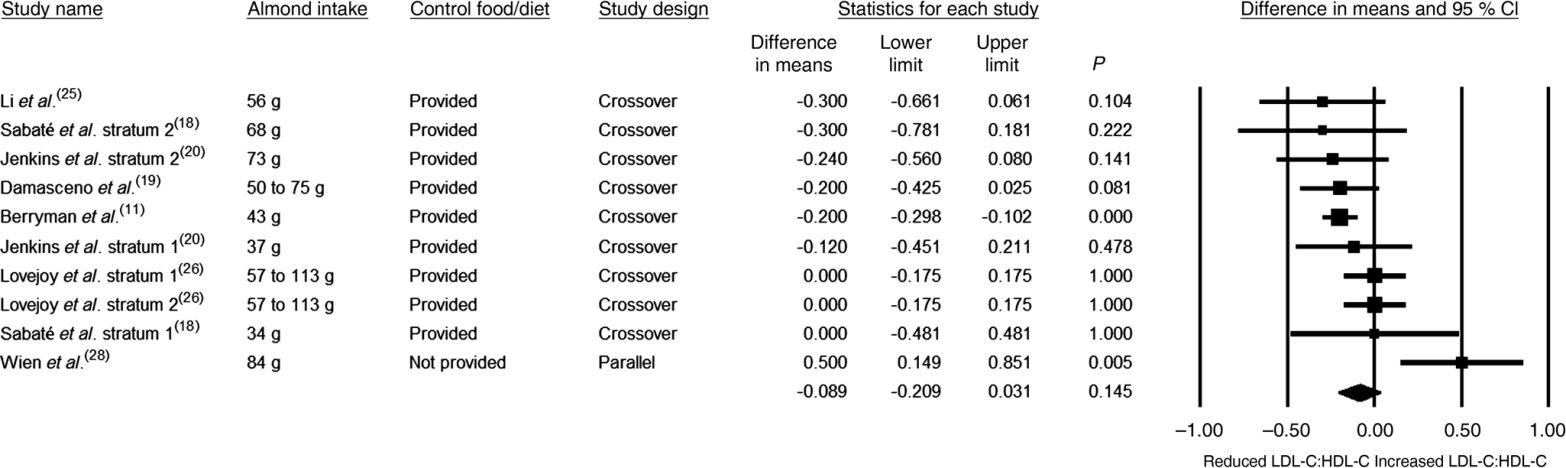
Effect of almond consumption on the ratio of LDL-cholesterol:HDL-cholesterol (LDL-C:HDL-C).

With regards to the ratio of TC:HDL-C, when data from all nine strata were pooled, the reduction in the ratio was statistically significant (see Table 3 and Fig. 6). However, publication bias was detected. Using trim and fill, two studies were found to be missing to the right of the pooled effect size, and with these studies imputed, the pooled effect, though negative (i.e. favourable), was smaller and no longer statistically significant (Table 3). Results for the subgroup analyses were in the same direction of effect (i.e. the pooled effect was negative), with variable statistical significance. With regards to the ratio of LDL-C:HDL-C, when data from all ten strata were pooled, the reduction in the ratio was not significant (−0·089; 95 % CI −0·209, 0·031; *P* = 0·145) (see Table 3 and Fig. 7). As for the ratio of TC:HDL-C, publication bias was detected, and using trim and fill, two studies were found to be missing to the right of the pooled effect size for LDL-C:HDL-C. With these studies imputed, the pooled effect, though negative (i.e. favourable), was smaller and remained non-statistically significant (Table 3). Results for the subgroup analyses were in the same direction of effect (i.e. the pooled effect was negative), with variable statistical significance.

## Discussion

In a meta-analysis that included five randomised control trials (and nine strata), Phung *et al*.^(^^7^^)^ reported that almonds significantly reduce TC and have a strong trend towards reducing LDL-C (*P* = 0·05). Although Phung *et al*.^(^^7^^)^ also reported a near-significant reduction in HDL-C (*P* = 0·08) and no effect on TAG, in our analyses, which are based on a total of eighteen publications and twenty-seven strata, the intake of almonds was associated with significant reductions in TC, LDL-C and TAG, and no effects on HDL-C.

In a meta-analysis and dose–response of sixty-one controlled intervention trials, which ranged in duration from 3 to 26 weeks, the consumption of nuts was associated with significant reductions in TC, LDL-C, apoB and TAG, with greater effects observed with a nut intake of 60 g/d and in individuals with T2DM^(^^29^^)^. In contrast, in a Cochrane review, Martin *et al.*^(^^30^^)^ reported that the intake of nuts had no effects on LDL-C or HDL-C (for TC and TAG, substantial heterogeneity precluded the pooling of results). The Cochrane assessment was based only on three publications (and four strata): Tey *et al.*^(^^31^^)^ (who provided 42 g of hazelnuts to generally healthy male and female adults for 12 weeks); Abazfarad *et al.*^(^^9^^)^ (who provided 50 g of almonds to overweight and obese premenopausal women for 3 months); and Tey *et al.*^(^^32^^)^ (who provided 30 or 60 g of hazelnuts to overweight and obese male and female adults for 12 weeks). The main objective of the Cochrane review was to assess the effects of nut consumption on the primary prevention of CVD. In none of the studies was the incidence of heart disease assessed; thus, the effects of nut consumption on surrogate measures of CVD risk were examined. With such few studies, and with three of the four strata conducted in generally healthy subjects, it is no surprise that effects on blood lipid levels could not be identified.

Based on our systematic evidence-based review and meta-analyses, which included a total of eighteen publications and twenty-seven strata, the intake of almonds was associated with significant reductions in TC, LDL-C and TAG, and no effects on HDL-C. In all of the included studies, almonds or diets enriched with almonds were provided to the subjects; however, the control was variable across the studies. In thirteen of the twenty-seven strata, a control food or diet was not administered to the subjects and the subjects were instructed not to consume nuts. In fourteen of the twenty-seven strata, the subjects were provided with a control food or a control diet. It seems that almonds effectively improve TC and LDL-C, whether the comparison is made with the consumption of no almonds or with a control food or diet that, at the very least, was isoenergetic to the almond intervention. Based on the other subgroup analyses, it seems that the efficacy of almonds in improving TC and LDL-C is greatest with a daily almond intake of 45 g or more and in individuals whose TC and LDL-C levels at baseline are elevated (i.e. not optimal). Although pooling the results of the crossover studies (but not the parallel studies) resulted in a significant reduction in LDL-C, the results should be interpreted with caution, given that the crossover strata were comprised predominantly of subjects whose baseline LDL-C levels were not optimal, while the parallel strata were comprised predominantly of subjects whose baseline LDL-C levels were optimal. Likewise, although pooling the results of the studies with a duration <12 weeks (but not the studies with a duration ≥12 weeks) resulted in significant reductions in both TC and LDL-C, the results should be interpreted with caution, given that there were only six strata with a duration ≥12 weeks, and in three of these six strata, the intake of almonds was <45 g/d and/or the subjects had optimal levels of TC and/or LDL-C at baseline^(^^10^^,^^16^^,^^24^^)^. Of the three parallel strata that were 12 weeks or longer in duration and in which the almond intake was ≥45 g/d and the baseline lipid levels were not optimal, there were significant or near-significant reductions in both TC and LDL-C in two of the strata^(^^9^^,^^23^^)^.

There is preliminary evidence that the consumption of almonds also leads to favourable changes in the ratio of TC:HDL-C; however, this lipid parameter was assessed only in nine strata, and the improvement was no longer statistically significant once an adjustment for publication bias was made. Preliminary evidence that the consumption of almonds leads to favourable changes in the ratio of TC:HDL-C is consistent with our findings of significant reductions in TC, with no effects on HDL-C. LDL-C as well as the ratio of TC:HDL-C are recognised as surrogate measures of CHD risk. Thus, it is plausible that by improving the blood lipid profile, the consumption of almonds would also be associated with significant reductions in the risk of CHD. While an intervention study on the effects of almonds on the risk of CHD has yet to be conducted, there is evidence from both prospective observational studies and a randomised controlled trial that the consumption of nuts, in general, is associated with significant reductions in the incidence of heart disease (discussed in the following paragraph).

In a meta-analysis of thirteen prospective studies (involving a total of 347 477 individuals and 6127 cases of coronary artery disease (CAD)), the relative risk (RR) of CAD was significantly reduced with the highest *v.* the lowest consumption of nuts (RR 0·660; 95 % CI 0·581, 0·748); moreover, the protective effect of nuts against the development of CAD was found to be dose-dependent, such that risk decreased by 5 % for every additional serving of nuts consumed per week^(^^33^^)^. In the PREDIMED (PREvención con DIeta MEDiterránea) study, which is a large, multi-centre primary prevention trial of the effects of three diets on CVD risk, the consumption of a Mediterranean diet supplemented with either extra-virgin olive oil or nuts resulted in significant reductions in CVD cases (including cases of myocardial infarction, stroke, or CVD death) relative to a control group instructed to consume a diet low in fat^(^^34^^)^. In a recent cross-sectional study involving 3 312 403 Americans undergoing screening for peripheral arterial disease, those who consumed nuts every day were 21 % less likely to have peripheral arterial disease relative to those who consumed nuts less than once per month; this statistically significant finding was evident even after adjusting for several important variables, such as age, sex, smoking status, obesity, family history of CVD, diet and the presence of diet-related diseases such as diabetes^(^^35^^)^.

The mechanism by which the consumption of nuts leads to favourable alternations in blood lipid levels is not fully understood. Nuts are nutrient dense, have a favourable fatty acid profile, and contain other constituents such as sterols and flavonoids that, collectively, may be important in the mechanism of almonds in improving blood lipid levels and CHD risk. In addition, it is possible that the favourable changes in blood lipid levels with the consumption of almonds are related, at least in part, to concomitant improvements in body weight and body composition. In several of the studies that were included in our meta-analysis, there were significant reductions in body weight with the consumption of almonds relative to the control^(^^11^^,^^20^^,^^28^^)^. The study by Berryman *et al.*^(^^11^^)^ is of particular interest, given that the subjects were provided with all of their foods during both the almond and control intervention periods, and the diets were rigorously controlled. There were statistically significant improvements in body weight, waist circumference and body composition (including abdominal fat mass) with the 6-week consumption of the almond diet relative to the control diet. Recently, it was demonstrated that the energy value of almonds calculated using the Atwater factors is 32 % greater than the actual energy that is metabolisable from almonds^(^^36^^)^. Similar observations have also been made for pistachios and walnuts^(^^37^^,^^38^^)^. This could explain the reductions in body weight that have been observed in some of the studies with the consumption of almonds. If not all of the ‘calculated’ energy in almonds is actually metabolisable, then in highly controlled experimental studies where the diets are prepared and provided to the study participants (such as in the study by Berryman *et al.*^(^^11^^)^), the diets may not have been truly isoenergetic.

The consumption of nuts is encouraged in several ‘heart-healthy’ diets. Nuts are important constituents of the portfolio diet, which also consists of plant sterols, viscous fibres and soya protein^(^^39^^)^. Likewise, nuts are part of the Mediterranean diet, which consists also of fruits and vegetables, legumes, whole-grain cereals, olive oil, fish and seafood, herbs and spices, and moderate amounts of meat, dairy products and wine^(^^40^^)^. Nuts are constituents of the Palaeolithic diet, which also includes lean meat, fish, fruit, leafy and cruciferous vegetables, root vegetables and eggs^(^^41^^)^. The consumption of nuts, such as almonds, as part of a healthy diet should be encouraged in order to help in the maintenance of normal blood lipid levels and to reduce the risk of heart disease.
